# Irritability and Hormone Replacement Therapy in Women Attending a Menopause Clinic: A Cross-Sectional Study

**DOI:** 10.3390/jcm15207956

**Published:** 2026-10-14

**Authors:** Miriam Olivola, Andrea Agosta, Antonella Consonni, Roberta de Filippis, Francesca Regispani, Lucia Margherita Reina, Vassilis Martiadis, Gabriella Fantini, Barbara Sanna, Lucia Calò, Erika Ciocca, Roberta Anniverno, Bernardo Maria Dell’Osso

**Affiliations:** 1 Department of Mental Health and Addiction Services, Macedonio Melloni Hospital, ASST Fatebenefratelli-Sacco, 20129 Milan, Italy; 2 School of Industrial sEngineering, University Cattaneo (LIUC), 21053 Castellanza, Italy; 3 Intelligence, Complexity, and Technology Lab (ICT Lab), University Cattaneo (LIUC), 21053 Castellanza, Italy; 4 Department of Mental Health, Azienda Sanitaria Locale Napoli 1 Centro, 80125 Naples, Italy; 5 Department of Pharmacological and Biomolecular Sciences, University of Milan, 20133 Milan, Italy; 6 Department of Biomedical and Clinical Sciences “Luigi Sacco”, University of Milan, 20157 Milan, Italy; 7 “Aldo Ravelli” Center for Nanotechnology and Neurostimulation, University of Milan, 20142 Milan, Italy; 8 Department of Psychiatry and Behavioral Sciences, Stanford University, Stanford, CA 94305, USA; 9 Centro per lo Studio dei Meccanismi Molecolari alla Base delle Patologie Neuro-Psico-Geriatriche, University of Milan, 20129 Milan, Italy

**Keywords:** menopause, hormone replacement therapy, irritability, oxidative stress, affective symptoms, Greene Climacteric Scale

## Abstract

**Background**: The menopausal transition is frequently associated with affective symptoms, including irritability, which remains relatively understudied despite its clinical relevance. Hormone replacement therapy (HRT) may influence affective symptoms through hormonal and metabolic mechanisms, including modulation of oxidative stress. However, the relationship between HRT and irritability in menopausal women remains poorly understood. **Methods**: We conducted a cross-sectional study of 84 women attending a menopause clinic between 2020 and 2025. Irritability was assessed using the single irritability item of the Greene Climacteric Scale. Associations between HRT use and irritability were explored using descriptive statistics, multivariable logistic regression, and exploratory predictive modelling controlling for age and clinical variables. **Results**: Approximately 27% of participants were receiving HRT. Descriptive analyses suggested higher HRT use among women reporting irritability compared with those without irritability. However, multivariable models did not confirm a statistically significant association between irritability and HRT use (OR = 1.66, *p* = 0.244). Age was the only variable significantly associated with HRT use (OR = 0.89, *p* = 0.009). Exploratory differences in psychopharmacological prescribing patterns did not reach statistical significance. **Conclusions**: In this clinical sample, irritability was a common symptom during the menopausal transition but was not significantly associated with HRT use. The medication-class findings were exploratory and did not establish an association with irritability. Larger longitudinal studies are needed to clarify the relationship between hormonal treatments and affective symptoms in menopause.

## 1. Introduction

The menopausal transition represents a critical phase in a woman’s life, marked by endocrine, metabolic, and psychosocial changes. While vasomotor symptoms such as hot flashes and night sweats are widely recognized, affective symptoms may also affect quality of life and daily functioning. Longitudinal studies have documented increased vulnerability to depressive symptoms and major depression during this transition [1,2]. Irritability is another affective complaint that warrants consideration within the broader assessment of mood symptoms in midlife women [3].

Hormonal changes have been implicated in mood symptoms during the menopausal transition, and estradiol-based interventions have been investigated for the treatment and prevention of depressive symptoms [4,5]. Relationships between sex hormones, antioxidant status, oxidative stress, and psychological symptoms have also been reported [6,7,8]. These findings provide a theoretical background to the present study; oxidative stress and inflammatory biomarkers were not directly assessed.

Affective symptoms during the menopausal transition require assessment within the broader clinical and psychosocial context [3]. The Greene Climacteric Scale (GCS) includes irritability within its psychological domain [9]. This supports assessing irritability alongside other menopausal symptoms, without assuming that a single item identifies a distinct clinical phenotype.

Hormone replacement therapy (HRT) has also been investigated in relation to oxidative balance in postmenopausal women [10,11,12]. These studies concern biological outcomes and do not establish an effect on irritability. Although estrogen-based interventions have been studied in relation to depressive symptoms [4,5], their findings should not be directly extrapolated to irritability.

The present study aimed to investigate the association between HRT use and irritability in women attending a menopause clinic. Descriptive analyses and multivariable statistical modelling were complemented by exploratory analyses of symptom groupings and treatment patterns.

## 2. Methods

### 2.1. Study Design and Population

This cross-sectional study analyzed data from 84 patients attending the Menopause Clinic at Macedonio Melloni Hospital, Milan, Italy, between 2020 and 2025. Data were collected during the initial clinical visit (T0) and included sociodemographic information, medical history, current medications, and standardized symptom assessments. The study was a retrospective, cross-sectional secondary analysis of information collected during routine clinical care at the Menopause Clinic. The clinical assessments and questionnaires were part of the usual diagnostic and treatment pathway; no additional intervention, examination, or research-specific procedure was performed for this study. Written informed consent for publication of anonymized clinical data was obtained from the patients in accordance with institutional procedures.

Women were included if they presented to the menopause clinic for an initial clinical assessment, were aged 40–75 years, and had a fully completed Greene Climacteric Scale on record. Women with incomplete GCS data were excluded. Participants were recruited consecutively through clinical referral over the study period (2020–2025).

Detailed information on HRT type (estrogen-only or combined estrogen–progestogen therapy), route of administration, and treatment duration was not available. HRT was therefore recorded as a binary use/non-use variable.

### 2.2. Assessment Instruments

The Greene Climacteric Scale (GCS) was administered to assess menopausal symptoms. It comprises 21 items covering psychological symptoms, with anxiety and depression components, somatic symptoms, vasomotor symptoms, and an additional item assessing loss of sexual interest [9]. Irritability is included within the psychological domain. Each item is rated on a 4-point scale (0 = not at all, 1 = a little, 2 = quite a bit, 3 = extremely), providing a dimensional assessment of symptom severity. For the main regression analysis, the irritability item was dichotomized as absent (score 0) or present (scores 1–3). This single-item measure and its dichotomization do not establish a validated clinical phenotype.

### 2.3. Statistical Analyses

Several statistical approaches were used to explore the relationship between HRT use and irritability, including descriptive statistics, logistic regression, and exploratory machine-learning approaches.

1. Descriptive Analysis: To observe raw patterns within the data regarding HRT distribution across irritability classes.
2. Inferential Modeling: Logistic regression to assess statistical support for observed patterns, controlling for potential confounders including age, premenstrual syndrome history, and pre-menopausal psychopathology. Bayesian network analysis (score-based algorithm, pgmpy) to explore probabilistic dependencies among five variables: HRT use, irritability (binary), age (centred), history of premenstrual syndrome, and pre-menopausal psychopathology. This analysis was conducted on an exploratory basis.
3. Predictive Modeling: K-means clustering (k = 2, determined using the elbow criterion) applied to normalized GCS subscale scores to identify latent patient groups with similar symptom profiles; cluster stability was not formally assessed and this analysis was conducted on an exploratory basis. Logistic classification with 70/30 train–test split to predict HRT usage.

Continuous variables were compared using Kruskal–Wallis tests, and categorical variables using chi-square or Fisher’s exact tests. Statistical significance was set at *p* < 0.05. All analyses were performed using Python 3.8 with scikit-learn, statsmodels, and pgmpy libraries.

Given the relatively small sample size, the machine learning analyses were considered exploratory and were primarily intended to identify potential patterns within the data rather than to build predictive clinical models. The Bayesian network, K-means clustering, and classification analyses were performed within the same small sample and should not be regarded as independent validation or evidence of robustness.

For the exploratory symptom-group analysis, women were classified according to the dominant affective presentation at the initial visit: the anxious group (*n* = 8) had predominant anxiety without clinically marked irritability; the depressed-without-irritability group (*n* = 6) had depressive features and a GCS irritability score of 0; and the irritable group (*n* = 51) had irritability as the dominant affective feature. Nineteen of the 84 women (22.6%) had mixed or ambiguous presentations that could not be assigned to a single dominant group and were excluded from this subgroup analysis, leaving 65 participants. These descriptive groups were separate from the K-means clusters and were not validated diagnostic categories.

Menopausal stage and vasomotor symptoms were not included as covariates in the primary logistic regression model owing to concerns about multicollinearity and insufficient cell sizes within subgroups; their potential role as confounders is acknowledged as a study limitation.

For the medication summaries, “multimodal” denoted vortioxetine, including the recorded combination with oxcarbazepine; it did not denote polypharmacy in general. The detailed “other” category included trazodone and agomelatine. “Phyto/other” denoted a supplement whose composition was not specified. In the multinomial regression the aggregated “other drugs” outcome comprised the study-coded mood-stabilizer, multimodal, and phyto/other categories; it was distinct from the detailed “other” category used in the descriptive summaries.

Combination regimens were assigned to a single recorded category: venlafaxine with lithium or lorazepam and duloxetine with delorazepam were coded as SNRIs; sertraline with alprazolam as an SSRI; vortioxetine with oxcarbazepine as multimodal; and perphenazine with amitriptyline as a tricyclic regimen. These classifications therefore represent the assigned regimen category rather than separate counts for each co-prescribed drug.

## 3. Results

### 3.1. Sample Characteristics

The sample comprised 84 women with a mean age of 58.4 ± 7.5 years (range: 42–70). Half of the participants (50%) were in early postmenopause, 41.7% were perimenopausal, and 8.3% were in late postmenopause. The mean age at menopause was 48.5 ± 10.9 years. A history of premenstrual syndrome was reported by 41.7% of participants. Most women were married or cohabiting (64.3%), employed (82.1%), and had completed at least secondary education (75.0%). Approximately 27% of the sample was using HRT at the time of assessment; by menopausal stage, HRT use was reported by [29%] of perimenopausal, [29%] of early postmenopausal, and [14%] of late postmenopausal women (Table 1).

### 3.2. Distribution of HRT Use by Irritability Class

**Figure 1:**
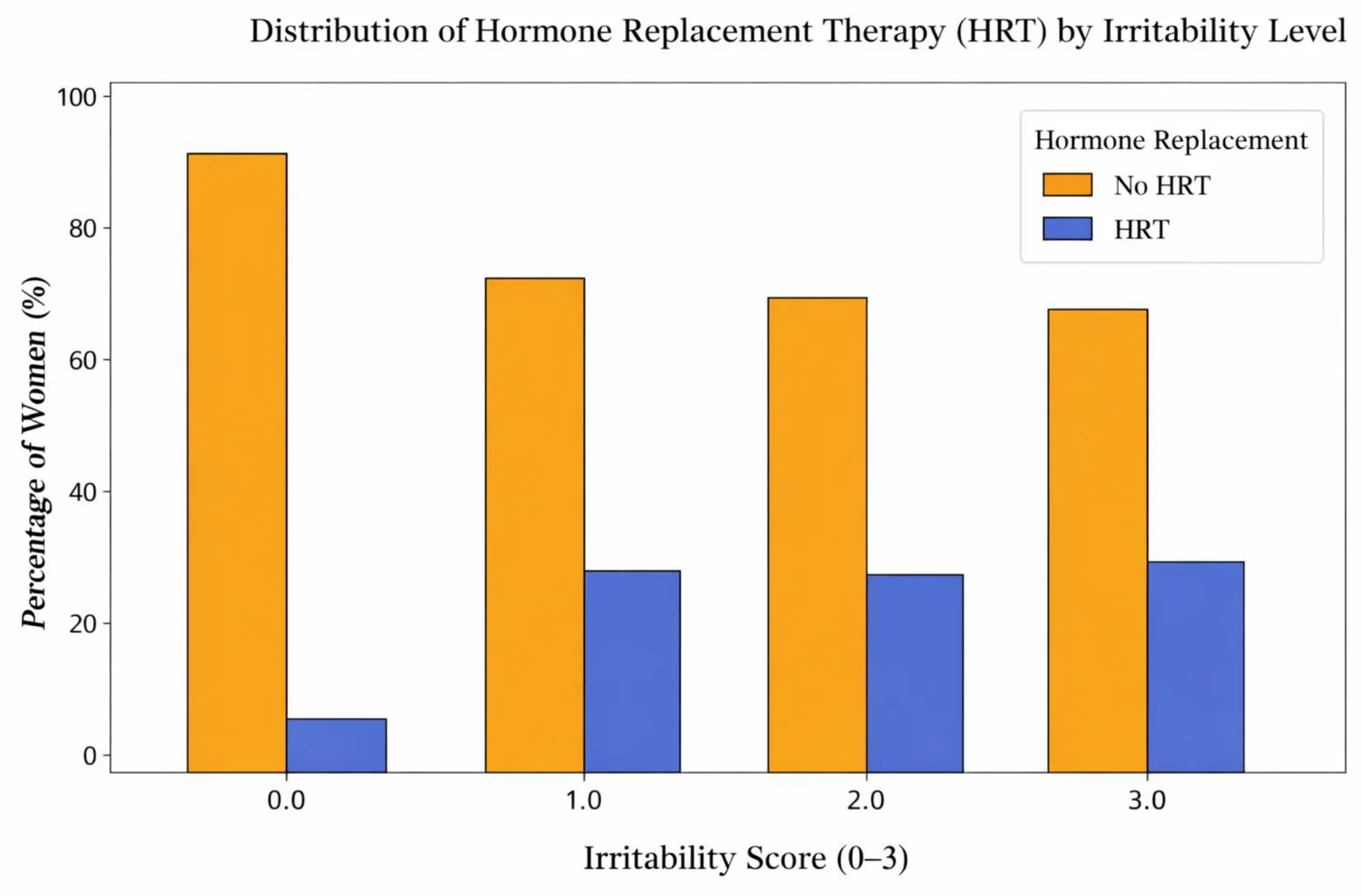
Distribution of HRT use by irritability class. Bar chart showing percentage of women using HRT (blue bars) versus not using HRT (orange bars) across four irritability classes (0 = none, 1 = mild, 2 = moderate, 3 = severe) based on the Greene Climacteric Scale.

Initial descriptive analyses indicated some variation in HRT use across levels of irritability (Figure 1). Among women who did not report irritability (score = 0), only 7.7% were receiving HRT. In contrast, roughly 30% of women reporting some degree of irritability (scores 1–3) were using HRT, with very similar proportions observed across mild (30%), moderate (31%), and severe (32%) irritability categories.

Because the overall sample size was modest and the number of HRT users relatively limited, irritability was entered into the regression models as a binary variable (absence vs. presence; score 0 vs. ≥1). This approach was chosen to improve model stability and reduce the impact of sparse data across categories. Additional sensitivity analyses were conducted treating irritability as an ordinal variable (0–3). These analyses yielded directionally consistent but unstable estimates with wider confidence intervals, reflecting sparse observations in the highest irritability categories. Taken together, the descriptive findings suggested a possible relationship between irritability and HRT use. However, this apparent pattern needed to be evaluated within multivariable models to determine whether it persisted after accounting for other clinical and demographic factors.

### 3.3. Inferential Analyses

**Figure 2:**
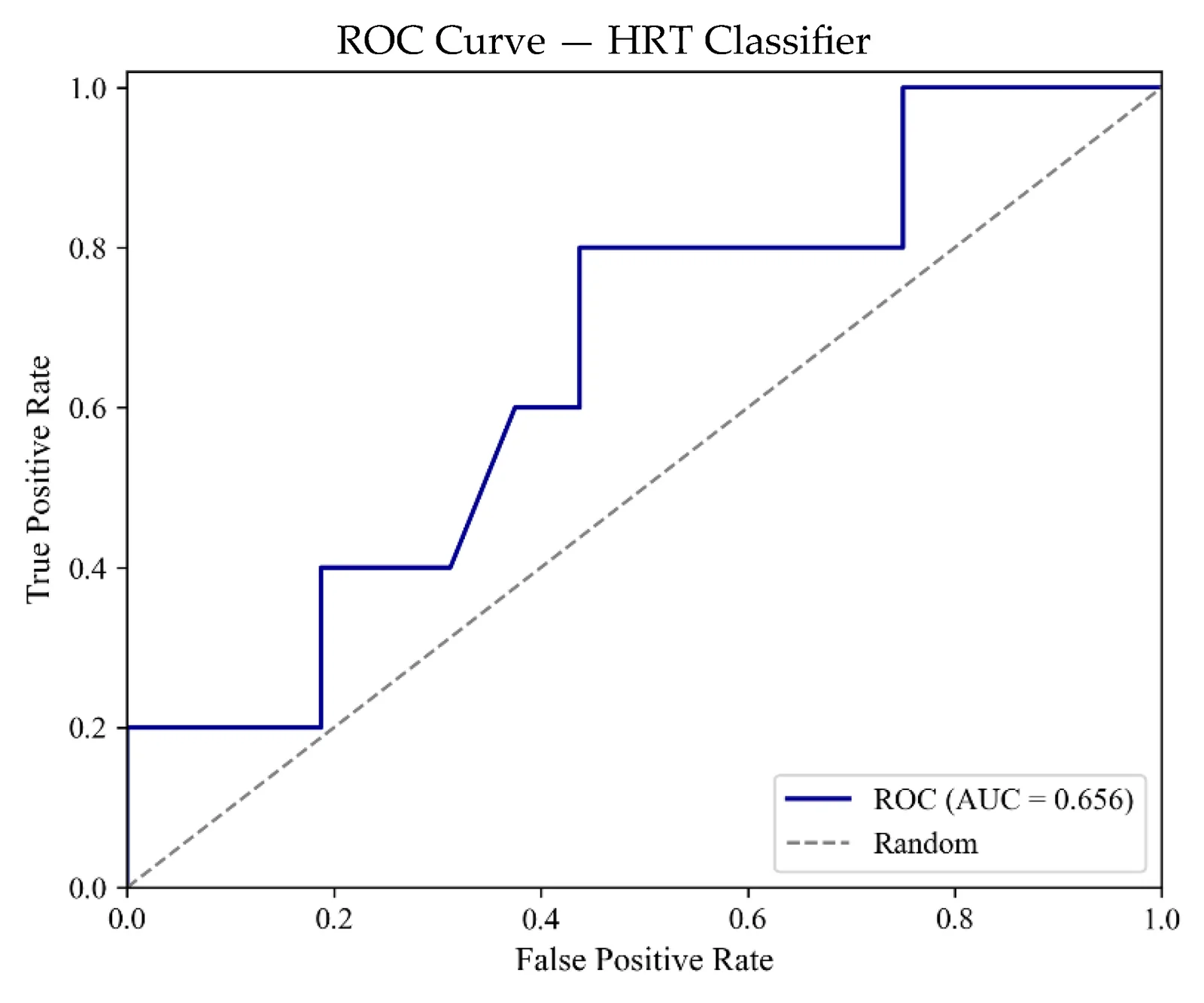
The ROC curve illustrates the discriminative ability of the logistic regression model predicting HRT use. The area under the curve (AUC = 0.656) indicates modest predictive performance, suggesting limited ability of age and irritability to accurately distinguish between HRT users and non-users.

**Figure 3:**
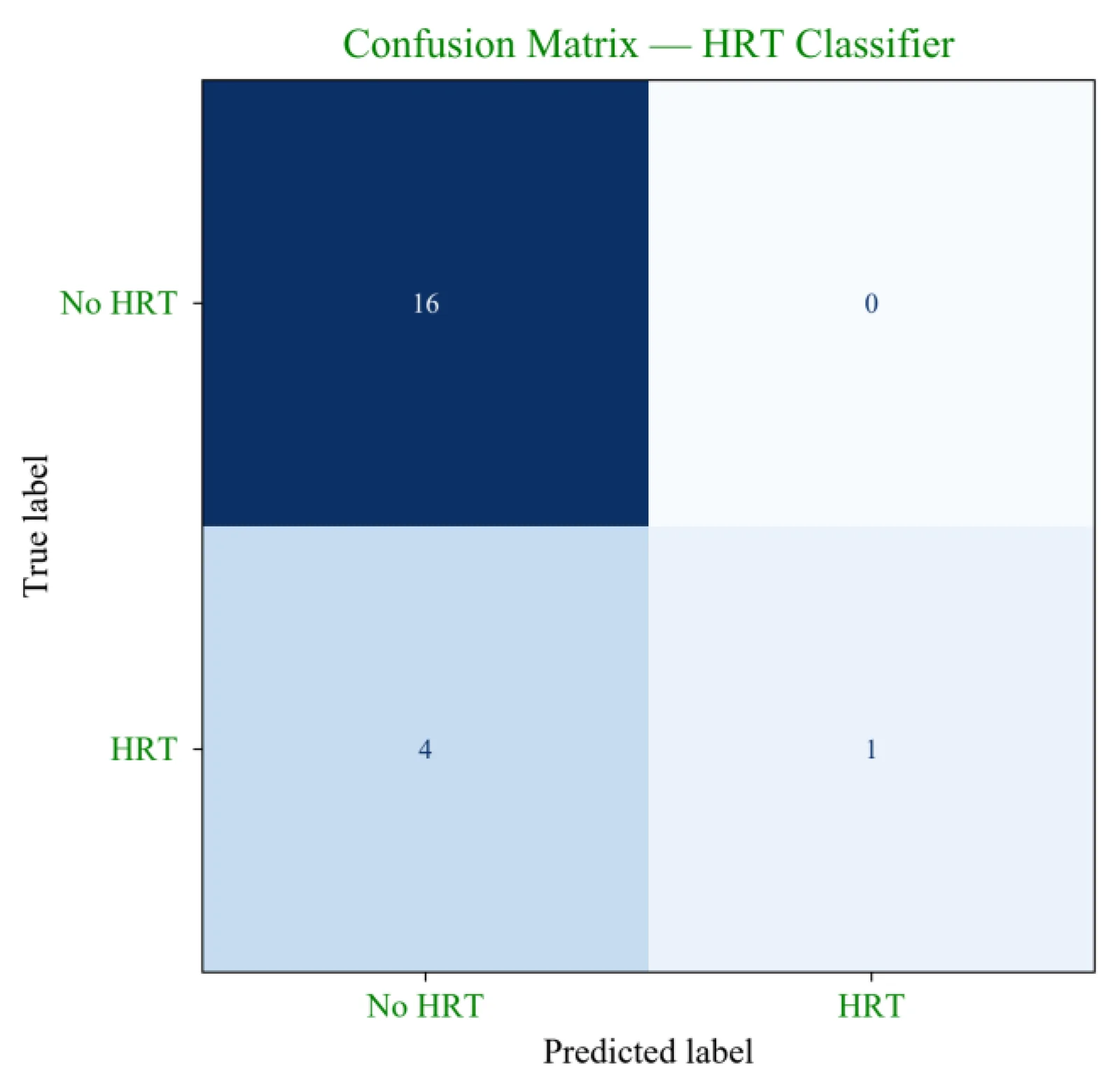
Confusion matrix of the logistic regression model evaluating HRT use in the test set. The model correctly classified 16 non-users and 1 user of HRT, with 4 users misclassified as non-users and no false positives. Only 1 of 5 HRT users was correctly identified (sensitivity = 20%), indicating limited ability to detect the minority class. Given the small sample size and class imbalance, these findings should be interpreted as exploratory and suggest limited classification utility. Blue shading represents the number of observations in each cell, with darker shades indicating higher counts. Green text is used for visual presentation only and has no statistical meaning.

**Figure 4:**
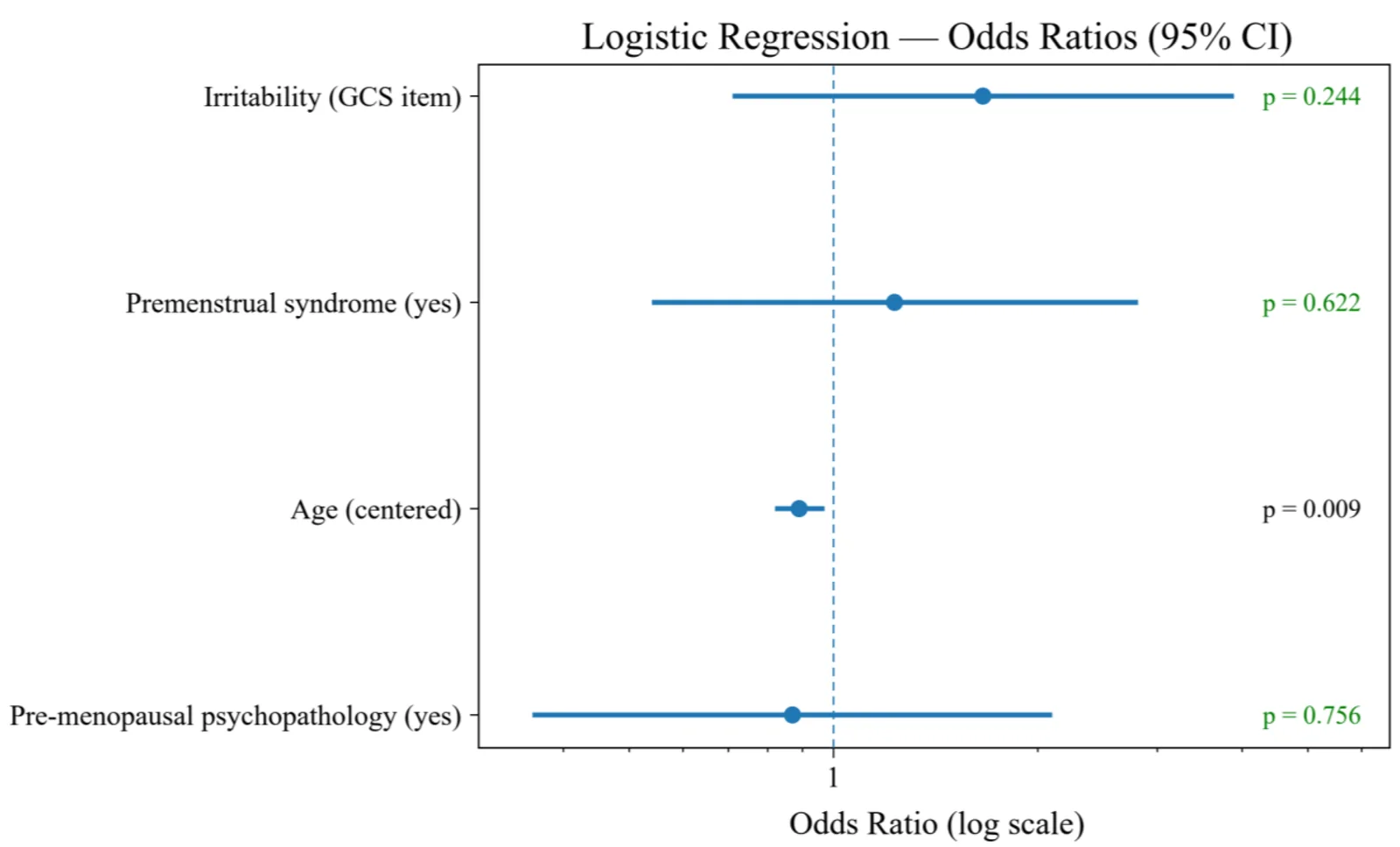
The multivariable logistic regression analysis showed that increasing age was significantly associated with reduced odds of HRT use (*p* = 0.009). Irritability, history of premenstrual syndrome, and pre-menopausal psychopathology were not significantly associated with HRT use, as their confidence intervals crossed the null value (OR = 1). The plotted ORs, 95% CIs, and *p*-values reproduce Table 2: *p* = 0.244 for irritability, *p* = 0.622 for premenstrual syndrome, and *p* = 0.756 for pre-menopausal psychopathology. Blue points represent odds ratios, and horizontal blue lines indicate their 95% confidence intervals. The dashed vertical blue line marks the null value (OR = 1). The colors of the *p*-value annotations have no statistical meaning; statistical significance is determined by the reported *p*-values.

Despite the suggestive descriptive pattern, logistic regression analysis did not confirm a statistically significant relationship between irritability and HRT use (OR = 1.66, 95% CI 0.71–3.89, *p* = 0.244) (Table 2). The only significant predictor of HRT use was age, with each additional year associated with an 11% decrease in the odds of HRT use (OR = 0.89, 95% CI 0.82–0.97, *p* = 0.009). Neither premenstrual syndrome history nor pre-menopausal psychopathology was significantly associated with HRT use (Figure 2, Figure 3 and Figure 4).

Bayesian network analysis of the five principal variables (HRT use, binary irritability, age [centred], history of premenstrual syndrome, and pre-menopausal psychopathology), using a score-based algorithm implemented in pgmpy, did not identify a direct connection between HRT and irritability in the fitted network. The conditional probability distributions of HRT use given different irritability levels were nearly identical. These exploratory findings from a small sample do not establish probabilistic independence.

### 3.4. Pharmacological Treatment Patterns

In the exploratory multinomial logistic regression, women with higher irritability scores showed a numerical tendency to receive anxiolytics rather than antidepressants (OR = 13.72, *p* = 0.070), or other medications rather than antidepressants (OR = 3.64, *p* = 0.052). Neither comparison reached conventional statistical significance. These findings should be interpreted as exploratory trends, rather than evidence that irritability predicts pharmacological class prescription (Table 3).

K-means clustering identified two exploratory symptom profiles: Cluster 0 (high-symptom profile) with elevated scores across all GCS subscales (normalized scores 0.55–0.94) received more diverse pharmacological treatments including benzodiazepines, mood stabilizers, and multimodal agents; Cluster 1 (low-symptom profile) with milder symptoms (normalized scores 0.22–0.47) was predominantly treated with SSRIs (Table 4). These groupings do not establish stable clinical subtypes.

**Figure 5:**
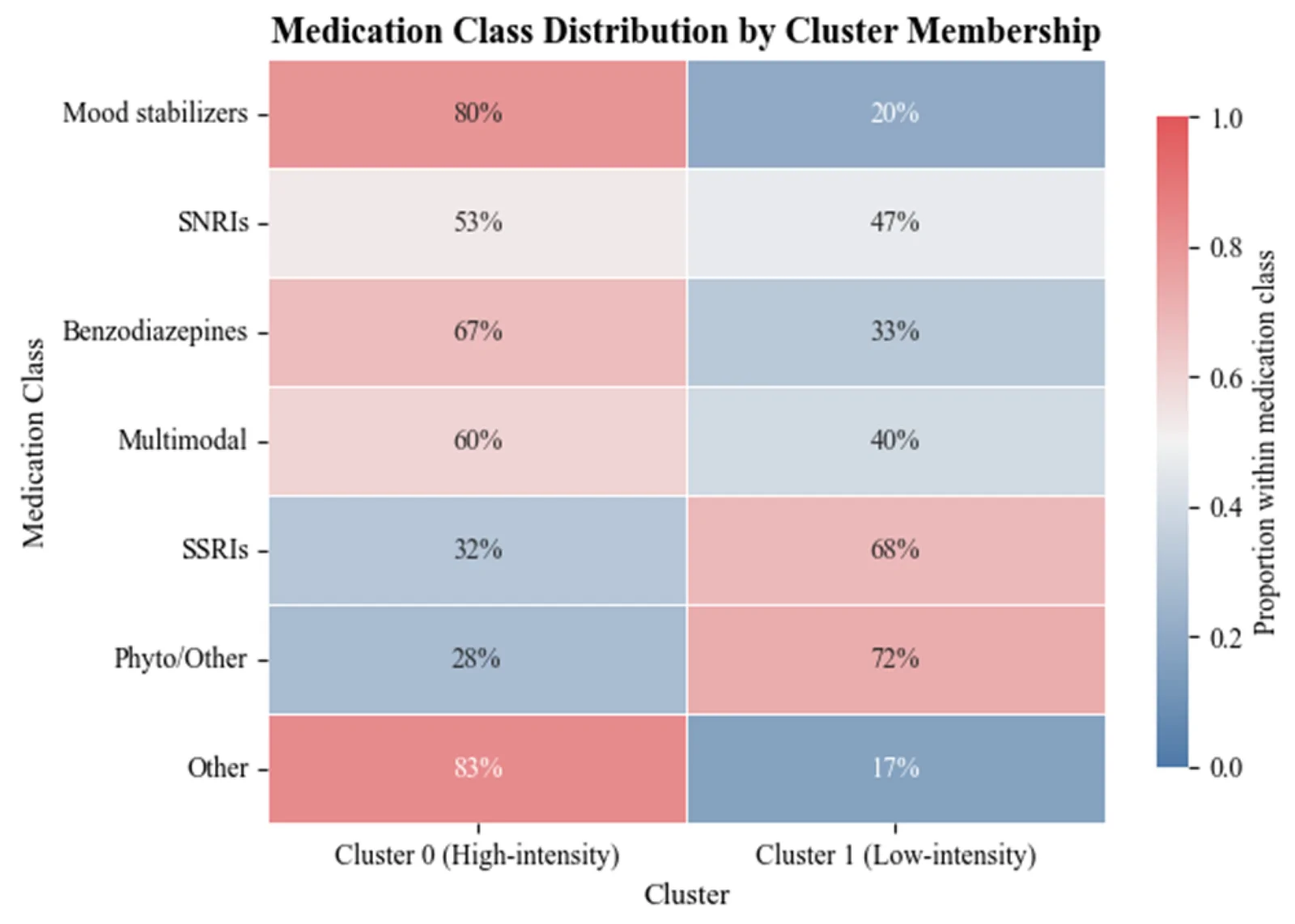
Medication class distribution according to exploratory K-means cluster membership.

Cross-tabulation of cluster membership with medication classes revealed exploratory descriptive patterns (Table 5). High-intensity patients were more frequently treated with mood stabilizers, benzodiazepines, multimodal agents, and other medications. In contrast, SSRIs and phyto/other treatments were predominantly prescribed in the low-intensity cluster. These descriptive patterns do not establish differential prescribing strategies; formal statistical testing was limited by small cell sizes (Figure 5).

Additional exploratory analyses examined whether the three descriptive symptom groups identified in the sample (anxious, depressed without irritability, and irritable) differed in terms of sociodemographic characteristics and selected clinical risk factors.

As shown in Table 6, no statistically significant differences were observed across groups for the examined sociodemographic variables. Mean age was slightly higher in the anxious group (61.75 ± 8.08 years) compared with the depressed without irritability group (55.50 ± 5.01 years) and the irritable group (57.80 ± 7.03 years), although this difference did not reach statistical significance (*p* = 0.245).

Similarly, the number of children did not significantly differ across groups, although women in the anxious group reported no children on average, while the other groups reported approximately one to one and a half children (*p* = 0.150). Age at menopause was also comparable across groups (*p* = 0.849). Employment rates were numerically higher among women in the irritable group (74.5%) compared with the anxious (62.5%) and depressed without irritability (50.0%) groups, although these differences were not statistically significant (*p* = 0.131). Overall, these findings suggest that the three descriptive symptom groups were broadly comparable in terms of sociodemographic background.

Clinical risk factors across groups are summarized in Table 7. No statistically significant differences were observed for the examined clinical variables. Nevertheless, some descriptive trends were noted. A history of premenstrual syndrome appeared more frequently in the irritable group (51.2%) compared with the anxious (28.6%) and depressed without irritability (20.0%) groups, although this difference did not reach statistical significance (*p* = 0.262).

Similarly, pre-menopausal psychopathology was numerically more common among women in the irritable group (45.1%) compared with the anxious (37.5%) and depressed without irritability (16.7%) groups (*p* = 0.397). Stressful life events during the previous year were reported by all participants in the depressed without irritability group (100%), compared with approximately half of participants in the anxious and irritable groups (both 50%), although this difference also did not reach statistical significance (*p* = 0.165).

Rates of current antidepressant use were relatively similar across groups, ranging from 62.5% in the anxious group to 70.6% in the irritable group (*p* = 0.890). Likewise, the proportion of women receiving hormone replacement therapy did not differ significantly between groups (*p* = 0.726).

Finally, differences in the receipt of psychological support were observed across symptom groups, with higher rates among women in the depressed without irritability group (75.0%) compared with the anxious (50.0%) and irritable (23.3%) groups. This difference did not reach the predefined threshold for statistical significance (*p* = 0.090).

Overall, these exploratory analyses suggest that the descriptive symptom groups identified in the sample were not clearly distinguished by the examined sociodemographic or clinical risk factors. However, some descriptive trends—particularly regarding premenstrual syndrome, pre-menopausal psychopathology, and psychological support—may warrant further investigation in larger samples.

## 4. Discussion

### 4.1. Principal Findings

The present study explored the relationship between hormone replacement therapy (HRT) use and irritability in women attending a specialized menopause clinic. Although initial descriptive analyses suggested that women reporting irritability were somewhat more likely to be receiving HRT, this association was not confirmed in multivariable statistical models. In particular, irritability did not emerge as an independent predictor of HRT use once potential confounding variables were taken into account.

Descriptive patterns may reflect demographic or clinical differences and should not be interpreted as evidence that irritability determines HRT prescribing decisions.

These findings should be considered in the context of recommendations emphasizing individualized assessment of menopausal symptoms and the benefits and risks of HRT. Treatment decisions take into account symptom burden, particularly vasomotor symptoms, medical history, contraindications, and patient preferences, rather than a single affective complaint [13,14].

### 4.2. Interpretation of the Exploratory Analyses

Logistic regression, Bayesian network modelling, and predictive classification provided complementary exploratory descriptions of this sample. Because they used the same small dataset, agreement between their results does not demonstrate robustness or independent validation.

The adjusted association between irritability and HRT use was not statistically significant (OR = 1.66, 95% CI 0.71–3.89, *p* = 0.244). The Bayesian network did not establish independence, and predictive discrimination was modest (AUC = 0.656). The classifier correctly identified only one of five HRT users in the test set, limiting its usefulness for identifying women receiving HRT.

Treatment decisions in menopausal care involve multiple biological, psychological, and clinical factors [15]. The exploratory models should therefore not be interpreted as clinically validated prediction tools.

### 4.3. Irritability as an Exploratory Affective Feature

The medication-class comparisons showed numerical trends for irritability, but neither the anxiolytic comparison (OR = 13.72, *p* = 0.070) nor the other-drug comparison (OR = 3.64, *p* = 0.052) reached statistical significance. These findings do not support describing irritability as the strongest predictor of prescribing.

Irritability should be considered within the broader assessment of mood symptoms, co-occurring psychiatric symptoms, and psychosocial factors during the menopausal transition [3,15]. These perspectives may inform future research, but the present single-item assessment cannot establish a distinct clinical phenotype or explain medication choices.

### 4.4. Oxidative Stress, Hormones, and Affective Symptoms

Changes in estrogen signalling and oxidative balance provide a theoretical context for affective symptoms during menopause [16]. However, relationships between hormonal changes and mood symptoms remain complex [17,18]. Because oxidative stress and inflammatory biomarkers were not measured in this study, our findings cannot test these biological mechanisms.

### 4.5. Clinical Implications

Irritability should be interpreted within the broader clinical and psychosocial presentation of women experiencing menopause [19]. In the present study, the nonsignificant medication-class comparisons cannot establish a prescribing preference or support treatment recommendations based on irritability alone.

Treatment decisions should continue to consider the overall symptom burden, psychological factors, medical comorbidities, and patient preferences [20]. Larger longitudinal studies are needed to clarify the clinical relevance of the exploratory patterns observed here.

## 5. Limitations

Several limitations should be considered when interpreting the findings of this study.

First, the cross-sectional design precludes causal inference regarding the relationship between HRT use and irritability. Longitudinal studies would be necessary to determine whether hormonal treatment influences irritability over time or whether baseline irritability affects decisions regarding HRT initiation or continuation.

Second, the relatively modest sample size (N = 84) may have limited the statistical power to detect small or moderate associations. Given the relatively low prevalence of HRT use in the sample, the resulting class imbalance may also have influenced the performance of predictive models.

Third, irritability was assessed using a single item from the Greene Climacteric Scale, which does not provide a multidimensional assessment of the construct. Dichotomization further reduces information about symptom severity. The symptom groups should therefore be regarded as exploratory descriptions rather than distinct or validated clinical phenotypes.

Furthermore, although ordinal sensitivity analyses were performed, the small number of observations within the higher irritability categories precluded stable ordinal modelling; these results should therefore be considered preliminary.

Fourth, the study did not include biological markers of oxidative stress or inflammatory activity, which limited the possibility of directly testing the proposed biological mechanisms linking hormonal changes and affective symptoms.

Another potential limitation concerns confounding by indication. In clinical practice, the decision to prescribe HRT is influenced by several factors—including vasomotor symptoms, cardiovascular risk profile, and patient preference—that were not fully captured in the present dataset.

In addition, detailed information on the type of HRT prescribed (estrogen-only versus combined estrogen–progestogen therapy), route of administration, and treatment duration was not available in the dataset. The HRT variable was therefore treated as a binary use/non-use indicator rather than as a pharmacologically characterized exposure, which limits interpretation of these findings.

Finally, because the study population consisted of women attending a specialized menopause clinic, the findings may not be fully generalizable to the broader population of menopausal women.

Finally, menopausal stage and vasomotor symptom burden could not be formally controlled in the primary regression model due to cell-size constraints; their potential confounding role in the relationship between irritability and treatment patterns cannot be excluded.

## 6. Conclusions

In this clinical sample, irritability was not significantly associated with HRT use after adjustment for the included covariates. Medication-class comparisons showed exploratory trends that did not reach statistical significance, and the single-item assessment does not establish a distinct irritability phenotype. Larger longitudinal studies are needed to clarify these relationships.

## Figures and Tables

**Table 1:** Sociodemographic Characteristics of the Sample (N = 84).

Variable	Value
Age (years)	
Mean ± SD	58.4 ± 7.5
Range	42–70
Menopausal status, n (%)	
Perimenopausal	35 (41.7%)
Early postmenopause	42 (50.0%)
Late postmenopause	7 (8.3%)
Age at menopause (years), Mean ± SD	48.5 ± 10.9
History of premenstrual syndrome, n (%)	35 (41.7%)
Marital status, n (%)	
Married/Cohabiting	54 (64.3%)
Single	19 (22.6%)
Separated/Divorced	10 (11.9%)
Widowed	1 (1.2%)
Living alone, n (%)	15 (17.9%)
Employed, n (%)	69 (82.1%)
Educational attainment, n (%)	
University degree or higher	28 (33.3%)
Secondary education	35 (41.7%)

**Table 2:** Logistic Regression Analysis: Predictors of HRT Use.

Predictor	OR	95% CI	*p*
Centered Age	0.89	0.82–0.97	0.009 **
Irritability	1.66	0.71–3.89	0.244
Premenstrual Syndrome	1.23	0.54–2.81	0.622
Pre-menopausal Psychopathology	0.87	0.36–2.10	0.756
Constant	0.31	0.12–0.81	0.017 *

Note: ** *p* < 0.01, * *p* < 0.05. OR = odds ratio; CI = confidence interval. HRT = Hormone Replacement Therapy.

**Table 3:** Exploratory Multinomial Logistic Regression: Correlates of Pharmacological Class Prescription.

Comparison/Predictor	OR	95% CI	*p*
Anxiolytics vs. Antidepressants			
Irritability	13.72	0.82–229.8	0.070
Anxiety	0.67	0.42–1.07	0.095
Sexuality	1.89	0.96–3.71	0.065
Other Drugs vs. Antidepressants			
Irritability	3.64	0.98–13.52	0.052
Anxiety	0.88	0.63–1.23	0.456
Vasomotor symptoms	1.12	0.75–1.67	0.578

Note: Reference category = Antidepressants (SSRIs/SNRIs). OR = odds ratio; CI = confidence interval.

**Table 4:** K-means Clustering: Symptom Profiles (Normalized GCS Scores).

GCS Subscale	Cluster 0 (High Symptom)	Cluster 1 (Low Symptom)
Anxiety	0.74	0.42
Depression	0.94	0.47
Vasomotor	0.55	0.22
Somatic	0.76	0.38
Sexual	0.82	0.44

Note: Scores normalized to 0–1 scale. Higher values indicate more severe symptoms. GCS = Greene Climacteric Scale.

**Table 5:** Pharmacological Treatment Distribution by Cluster.

Drug Class	Cluster 0 (n, %)	Cluster 1 (n, %)
SSRIs	8 (32%)	18 (58%)
SNRIs	4 (16%)	6 (19%)
Benzodiazepines	6 (24%)	3 (10%)
Mood Stabilizers	3 (12%)	2 (6%)
Multimodal	3 (12%)	1 (3%)
Other	1 (4%)	1 (3%)

Note: Cluster 0 (high-symptom profile) received more diverse treatments; Cluster 1 (low-symptom profile) predominantly received SSRIs.

**Table 6:** Sociodemographic Characteristics by Exploratory Symptom Group.

Variable	Anxious (*n* = 8)	Depressed w/o Irritability (*n* = 6)	Irritable (*n* = 51)	*p*
Age (years), M ± SD	61.75 ± 8.08	55.50 ± 5.01	57.80 ± 7.03	0.245
Number of children, M ± SD	0.00 ± 0.00	1.50 ± 1.00	1.30 ± 0.95	0.150
Age at menopause, M ± SD	50.00 ± 3.42	48.83 ± 1.47	47.43 ± 14.86	0.849
Employed, %	62.5	50.0	74.5	0.131

Note: M = Mean, SD = Standard Deviation. No significant differences between groups.

**Table 7:** Clinical Risk Factors by Exploratory Symptom Group (%).

Risk Factor	Anxious	Depressed w/o Irritability	Irritable	*p*
Premenstrual Syndrome	28.6	20.0	51.2	0.262
Psychopathology in Pregnancy/Postpartum	14.3	33.3	27.1	0.708
Psychiatric Family History	50.0	66.7	54.9	0.816
Pre-menopausal Psychopathology	37.5	16.7	45.1	0.397
Stressful Events (Past Year)	50.0	100.0	50.0	0.165
Current Antidepressant	62.5	66.7	70.6	0.890
HRT Use	25.0	16.7	31.4	0.726
Psychological Support	50.0	75.0	23.3	0.090

Note: Values are percentages. No factors reached statistical significance, though psychological support showed a trend (*p* = 0.090).

## Data Availability

The datasets generated and analyzed during the current study are available from the corresponding author on reasonable request.
